# Modeling and simulation of hypothermia effects on cardiac electrical dynamics

**DOI:** 10.1371/journal.pone.0216058

**Published:** 2019-05-03

**Authors:** Youssef Belhamadia, Justin Grenier

**Affiliations:** 1 Department of Mathematics and Statistics, American University of Sharjah, Sharjah, United Arab Emirates; 2 Department of Biomedical Engineering, University of Alberta, Edmonton, Canada; University of Minnesota, UNITED STATES

## Abstract

Previous experimental evidence has shown the effect of temperature on the action potential duration (APD). It has also been demonstrated that regional cooling of the heart can prolong the APD and promote the termination of ventricular tachycardia. The aim of this study is to demonstrate the effect of hypothermia in suppressing cardiac arrhythmias using numerical modeling. For this purpose, we developed a mathematical model that couples Pennes’ bioheat equation and the bidomain model to simulate the effect of heat on the cardiac action potential. The simplification of the proposed heat–bidomain model to the heat–monodomain model is provided. A suitable numerical scheme for this coupling, based on a time adaptive mesh finite element method, is also presented. First, we performed two-dimensional numerical simulations to study the effect of heat on a regular electrophysiological wave, with the comparison of the calculated and experimental values of *Q*_10_. Then, we demonstrated the effect of global hypothermia in suppressing single and multiple spiral waves.

## 1 Introduction

Several experimental studies have demonstrated the significant effect of induced hypothermia on cardiac and neurological outcomes for patients (see [1] for a review). Hypothermia is now recommended as a therapeutic treatment for cases of spinal cord and brain injuries (see [2] and [3]), and it is used as a standard treatment for cardiac arrest [4]. Numerical modeling can provide valuable contribution for the understanding of the role of temperature effects in the cardiac electrical dynamics, which is the main aim of this paper.

In literature, the modeling of the effects of temperature on the cardiac electrical wave has previously been performed mostly by modifying the ionic activity. For cells, an ionic model that considers the temperature dependence of electrical parameters was presented in [5]. It has been shown that the variations in the cellular responses due to changes in temperature can have profound effects on the behavior of the transmembrane potential, including action potential durations (APDs) (see [6] and [7]). In 2006, a modified FitzHugh–Nagumo monodomain model combined with the Pennes’ equation was proposed [8] to include the influence of temperature on the behavior of a simulated nerve. In 2009, this work was extended [9] by including the effect of temperature in the Hodgkin–Huxley model to further increase the accuracy of the description of this behavior. Similarly, temperature dependence was added to ionic intestine models [10] to estimate its possible effects during surgery.

Considering the cardiac dynamics, a cell model has recently been developed [11, 12] that includes the influence of temperature on ventricular electrical activity. Regional cooling has been studied in different tissue sizes [12] and it has been shown that it can be suitable as an anti-arrhythmic therapy for small tissue sizes and pro-arrhythmic therapy for large tissue sizes. However, the effect of temperature has been included only in ionic cardiac activity. To the best of our knowledge, the effect of global hypothermia on the cardiac electrophysiological wave, which is the main contribution of this paper, has not been previously reported in literature. Therefore, based on the idea of Bini and coworkers [8, 9, 11, 12], we have developed a mathematical model that combines Pennes’ bioheat equation with the bidomain model. The simplification of the proposed heat–bidomain model to the heat–monodomain model is presented. In the proposed heat–bidomain model, heat source terms considering the Joule heating effects in the intra- and extracellular regions are added. These source terms induced by the Joule effect strongly depend on the gradient of the action potential, and in cardiac tissue, this potential has a steep slope during the de- and repolarization phases; thus, the simulation becomes challenging. Owing mostly to this challenge, the spatial temperature effects on cardiac tissue have not been previously studied. This difficulty, reported in [9], limits the generalization of the methodology to the case of cardiac tissue. Therefore, in this paper, we also present a time-dependent adaptive mesh algorithm to address this challenge. The main advantage of this method is that it can concentrate the spatial resolution along the areas with large gradients, enabling the simulation of the heat–bidomain coupling. In this paper, numerical simulations are presented to study the effect of temperature variations on a regular electrophysiological wave and to investigate the effect of hypothermia in suppressing cardiac arrhythmias.

This paper is organized as follows. Section 2 discusses the proposed mathematical models for the coupling of the heat with the bi- and monodomain models. The finite element discretization and the time-dependent adaptive algorithm is presented in section 3. Finally, multiple simulations are provided in the last section, to study the effects of temperature variations on the action potential in two-dimensional tissues.

## 2 Models

The bi and monodomain models are widely used in electrocardiology to simulate the spatial propagation of the transmembrane potential in the myocardium. These classical models do not consider the effect of temperature on the electrical wave; therefore, heat–bidomain and heat–monodomain couplings are required.

### 2.1 Heat–bidomain model

In the bidomain model, the cardiac muscle is considered as two separate domains, namely, the intracellular domain, which considers the electric potential inside the cell, and the extracellular domain, which considers the electric potential outside the cell. The relationship between the intra- and extracellular currents *i*_*i*_ and *i*_*e*_, respectively, the potentials are ohmic and given by

$$\begin{array}{c} J_{i}=-G_{i}\nabla \phi_{i}\;\text{and}\;J_{e}=-G_{e}\nabla \phi_{e}, \end{array}$$ (1)

where *ϕ*_*i*_ is the intracellular potential, *ϕ*_*e*_ is the extracellular potential, and ***G**_i_* and ***G**_e_* are the intra- and extracellular conductivity tensors, respectively. Assuming that the total current is conserved, the following elliptic equation can be written:

$$\begin{array}{c} \nabla \cdot (G_{i}\nabla \phi_{i}+G_{e}\nabla \phi_{e})=0. \end{array}$$ (2)

At each point of the myocardial tissue, the transmembrane potential is considered as the difference between the intra- and extracellular potentials, *V*_*m*_ = *ϕ*_*i*_ − *ϕ*_*e*_. For any biological membrane, the transmembrane current is the sum of the ionic and capacitive currents given by

$$\begin{array}{c} \chi C_{m}\frac{\partial V_{m}}{\partial t}+\chi I_{ion}=I_{m}, \end{array}$$ (3)

where *C*_*m*_ is the capacitance of the cell membrane, *χ* is the ratio of the membrane surface area to the volume, and according to the current conservation law, *I_m_* = ∇ · (***G**_i_*∇*ϕ_i_*).

The total ionic current across the membranes, *I*_*ion*_, depends on the ionic models. In this paper, we consider two simplified two-variable ionic models. One is the Mitchell–Schaeffer model [13], introduced in 2003, which is derived from the Fenton–Karma ionic model [14]. This model quantitatively reproduces the behavior of the ventricular action potential, and it has similar de- and repolarization isochrons to those obtained by the more complex cardiac ionic models. In this work, the parameters for this model are adjusted to have a fast upstroke, which enables this model to be suitable for our study. The other is the Aliev–Panfilov model [15], introduced in 1996, which can reproduce more realistic shapes of the cardiac action potential as well as the APD restitution characteristic observed in experiments. This model has widely been used for simulating specific types of cardiac arrhythmia, such as those characterized by rotating waves, which are also explored in this study. In this paper, we scaled both the Mitchell–Schaeffer and the Aliev–Panfilov models to obtain physiologically interpretable values, based on the following equations. The Mitchell–Schaeffer model is given by

$$\begin{array}{c} I_{ion}\left(V_{m},W\right)=(\frac{v_{amp}}{\tau_{in}})W(\frac{V_{m}-v_{rest}}{v_{amp}}{)}^{2}(\frac{V_{m}-v_{rest}}{v_{amp}}-1)+(\frac{V_{m}-v_{rest}}{\tau_{out}v_{amp}}), \end{array}$$

$$\begin{array}{c} F_{ion}\left(V_{m},W\right)=\{\begin{array}{ccc} \frac{1-W}{\tau_{open}} & \;\text{for}\; & \frac{V_{m}-v_{rest}}{v_{amp}}<v_{gate},\\ -\frac{W}{\tau_{close}} & \;\text{for}\; & \frac{V_{m}-v_{rest}}{v_{amp}}\geq v_{gate}. \end{array} \end{array}$$

The Aliev–Panfilov model is given by

$$\begin{array}{c} I_{ion}\left(V_{m},W\right)=(\frac{k}{v_{amp}^{2}})\left(V_{m}-v_{rest}\right)\left(V_{m}-v_{a}\right)\left(V_{m}-v_{peak}\right)+\left(V_{m}-v_{rest}\right)W, \end{array}$$

$$\begin{array}{c} F_{ion}\left(V_{m},W\right)=\frac{1}{12.9}(\epsilon_{0}+\frac{\mu_{1}W}{\frac{V_{m}-v_{rest}}{v_{amp}}+\mu_{2}})(-W-k(\frac{V_{m}-v_{rest}}{v_{amp}})(\frac{V_{m}-v_{rest}}{v_{amp}}-a-1)). \end{array}$$

Assuming that the transmembrane potential *V*_*m*_ is *ϕ*_*i*_ − *ϕ*_*e*_, the bidomain model considered in this paper is given by

$$\begin{array}{c} \left\{ \begin{array}{c} \chi C_{m}\frac{\partial V_{m}}{\partial t}-\nabla \cdot \left(G_{i}\nabla V_{m}\right)=\nabla \cdot \left(G_{i}\nabla \phi_{e}\right)-\chi I_{ion}\left(V_{m},W\right),\\ \nabla \cdot \left(\left(G_{i}+G_{e}\right)\nabla \phi_{e}\right)=-\nabla \cdot \left(G_{i}\nabla V_{m}\right),\\ \frac{\partial W}{\partial t}=F_{ion}\left(V_{m},W\right). \end{array}\right. \end{array}$$ (4)

The bidomain model is not capable of studying any temperature effects. To address this problem, we first consider Pennes’ bioheat equation [16]. This equation, first introduced in 1948, describes the transfer of heat in biological tissues, and it can be written as

$$\begin{array}{c} \rho c_{p}\frac{\partial T}{\partial t}=\nabla \cdot \left(k\nabla T\right)+b_{c}\left(T^{*}-T\right) \end{array}$$ (5)

where *k* is the thermal conductivity, *T* is the temperature of the tissue, T* is the temperature of the supplied arterial blood, *b*_*c*_ is the strength of the heat sink due to blood perfusion, *ρ* is the density of the tissue, and *c*_*p*_ is the heat capacity of the tissue. However, this equation does not consider the effect of the heat due to the propagation of the electrical wave across the tissue. This response can be included simply by considering the Joule effect (see [17] and [8]). In our work, we consider the heat generation rate per unit volume from an electric field in the intra- and extracellular regions *p*_*i*_ and *p*_*e*_, respectively, as

$$\begin{array}{c} p_{i}=J_{i}\cdot \left(-\nabla \phi_{i}\right)\;\;\text{and}\;\;p_{e}=J_{e}\cdot \left(-\nabla \phi_{e}\right). \end{array}$$ (6)

These equations are incorporated as internal source terms in Eq (5) as follows:

$$\begin{array}{c} \rho c_{p}\frac{\partial T}{\partial t}=\nabla \cdot \left(k\nabla T\right)+b_{c}\left(T^{*}-T\right)+p_{i}+p_{e}. \end{array}$$ (7)

Thus, using the expressions of *J*_*i*_ and *J*_*e*_ given in 1, the coupling between the heat transfer in the tissue and the transmembrane potential in the myocardium can be given by equation

$$\begin{array}{c} \rho c_{p}\frac{\partial T}{\partial t}=\nabla \cdot \left(k\nabla T\right)+b_{c}\left(T^{*}-T\right)+G_{i}\nabla \phi_{i}\cdot \nabla \phi_{i}+G_{e}\nabla \phi_{e}\cdot \nabla \phi_{e}. \end{array}$$ (8)

Nevertheless, the temperature also has a significant effect on the ionic behavior in the cells. The above ionic models still do not consider the effect of temperature; therefore, they need to be modified to accurately represent the effects of temperature variation on the APD. For this purpose, we refer to the original study by Hodgkin and Huxley [18], in which the effect of the temperature on the rate of change of the conductance variables is considered. Subsequent modifications were performed [6], and the linear changes of the ionic conductances with respect to temperature were also considered [7]. Based on these studies, the temperature properties can be added directly to the Mitchell–Schaeffer and Aliev–Panfilov models as

$$\begin{array}{c} I\left(V_{m},W,T\right)=\left(A\left(1+B\left(T-T_{a}\right)\right)\right)I_{ion}\left(V_{m},W\right), \end{array}$$ (9)

$$\begin{array}{c} F\left(V_{m},W,T\right)=\left(Q^{(T-T_{a})/10}\right)F_{ion}\left(V_{m},W\right), \end{array}$$ (10)

where *A*, *B*, and *Q* are constants and *T*_*a*_ is a reference temperature, which is 37 °C in the case of cardiac cells. Therefore, the proposed heat–bidomain model for the simulation of the effect of temperature on the cardiac tissue can be written as

$$\begin{array}{c} \left\{ \begin{array}{c} \chi C_{m}\frac{\partial V_{m}}{\partial t}-\nabla \cdot \left(G_{i}\nabla V_{m}\right)=\nabla \cdot \left(G_{i}\nabla \phi_{e}\right)-\chi I\left(V_{m},W,T\right),\\ \nabla \cdot \left(\left(G_{i}+G_{e}\right)\nabla \phi_{e}\right)=-\nabla \cdot \left(G_{i}\nabla V_{m}\right),\\ \frac{\partial W}{\partial t}=F\left(V_{m},W,T\right),\\ \rho c_{p}\frac{\partial T}{\partial t}=\nabla \cdot \left(k\nabla T\right)+b_{c}\left(T^{*}-T\right)+G_{i}\nabla \left(V_{m}+\phi_{e}\right)\cdot \nabla \left(V_{m}+\phi_{e}\right)+G_{e}\nabla \phi_{e}\cdot \nabla \phi_{e}. \end{array}\right. \end{array}$$ (11)

### 2.2 Heat–monodomain model

A widely applied method of reducing the computational time of the bidomain is to reduce the two-by-two set of partial differential Eq (4) to a scalar partial differential equation, resulting in the monodomain model. A similar technique can be used for the heat–bidomain model mentioned above. However, only the heat source due to Joule effect terms in Pennes’ bioheat Eq (8) needs to be considered in this study. Thus, we assume that the wavefront of the transmembrane potential is not fully curved (see [19], page 110); thus the sum of the intra- and extracellular current densities is required to be equal to zero:

$$\begin{array}{c} G_{i}\nabla \phi_{i}+G_{e}\nabla \phi_{e}=0\;\;\text{or}\;\;\left(G_{i}+G_{e}\right)\nabla \phi_{e}+G_{i}\nabla V_{m}=0. \end{array}$$ (12)

Therefore, the heat source term in Eq (8) can be expressed as

$$\begin{array}{c} G_{i}\nabla \left(V_{m}+\phi_{e}\right)\cdot \nabla \left(V_{m}+\phi_{e}\right)+G_{e}\nabla \phi_{e}\cdot \nabla \phi_{e}\\ =G_{i}\nabla V_{m}\cdot \nabla V_{m}+\left(G_{i}+G_{e}\right)\nabla \phi_{e}\cdot \nabla \phi_{e}+2G_{i}\nabla \phi_{e}\cdot \nabla V_{m}.\\ =G_{i}\nabla V_{m}\cdot \nabla V_{m}+G_{i}\nabla \phi_{e}\cdot \nabla V_{m}. \end{array}$$

Under the assumption of equal anisotropy ratios, *G*_*i*_ = λ*G*_*e*_ and from Eq (12) we can write,

$$\begin{array}{c} G_{i}\nabla \phi_{e}=\frac{-\lambda }{1+\lambda }G_{i}\nabla V_{m}, \end{array}$$

which gives

$$\begin{array}{c} G_{i}\nabla \left(V_{m}+\phi_{e}\right)\cdot \nabla \left(V_{m}+\phi_{e}\right)+G_{e}\nabla \phi_{e}\cdot \nabla \phi_{e}=\frac{1}{1+\lambda }G_{i}\nabla V_{m}\cdot \nabla V_{m}. \end{array}$$

Thus, the coupling of the heat–bidomain model in Eq (11) can be simplified as

$$\begin{array}{c} \left\{ \begin{array}{c} \chi C_{m}\frac{\partial V_{m}}{\partial t}-\nabla \cdot \left(G\nabla V_{m}\right)=-\chi I\left(V_{m},W,T\right),\\ \frac{\partial W}{\partial t}=F\left(V_{m},W,T\right),\\ \rho c_{p}\frac{\partial T}{\partial t}=\nabla \cdot \left(k\nabla T\right)+b_{c}\left(T^{*}-T\right)+G\nabla V_{m}\cdot \nabla V_{m}, \end{array}\right. \end{array}$$ (13)

where the conductivity tensor ***G*** can be considered as $\frac{1}{1+\lambda }G_{i}$.

Although the derivation of the heat–monodomain was presented, all our numerical simulations were performed using the heat–bidomain model. In addition, the assumption in Eq (12) is restricted to non-curved wavefronts, which is not universal. To overcome this limitation, we suggest, in the case of the bidomain model, the consideration of the Joule heat source generated by the membrane voltage, ***G**_i_*∇*V_m_* · ∇*V_m_*, instead of that generated by the intra- and extracellular regions, ***G**_i_*∇*ϕ_i_* · ∇*ϕ_i_* + ***G**_e_*∇*ϕ_e_* · ∇*ϕ_e_*. Therefore, the Pennes’ bioheat Eq (8) can be replaced by

$$\begin{array}{c} \rho c_{p}\frac{\partial T}{\partial t}=\nabla \cdot \left(k\nabla T\right)+b_{c}\left(T^{*}-T\right)+G_{i}\nabla V_{m}\cdot \nabla V_{m}. \end{array}$$ (14)

The numerical experiments [20] show that Eq (14) also provides appropriate results when combined with the bidomain model. For the sake of simplicity, in the following, the heat–bidomain model used for all numerical simulations is given by

$$\begin{array}{c} \left\{ \begin{array}{c} \chi C_{m}\frac{\partial V_{m}}{\partial t}-\nabla \cdot \left(G_{i}\nabla V_{m}\right)=\nabla \cdot \left(G_{i}\nabla \phi_{e}\right)-\chi I\left(V_{m},W,T\right),\\ \nabla \cdot \left(\left(G_{i}+G_{e}\right)\nabla \phi_{e}\right)=-\nabla \cdot \left(G_{i}\nabla V_{m}\right),\\ \frac{\partial W}{\partial t}=F\left(V_{m},W,T\right),\\ \rho c_{p}\frac{\partial T}{\partial t}=\nabla \cdot \left(k\nabla T\right)+b_{c}\left(T^{*}-T\right)+G_{i}\nabla V_{m}\cdot \nabla V_{m}. \end{array}\right. \end{array}$$ (15)

All parameters for both the heat–bidomain model in Eq (15) and its simplified heat–monodomain version in Eq (13) are determined in the next subsection, enabling these models to be more suitable for studying the effect of temperature on the cardiac transmembrane potential.

### 2.3 Model parameters setting

To study the effects of temperature on the cardiac action potential, first, we need to assign reasonable values to all the necessary constants in the heat–bidomain model in Eq (11). Specifically, these parameters need to be suitable to reproduce the realistic stiffness of the slope in the de- and repolarization phases of the cardiac transmembrane potential. This is crucial to this study, as the source term in the heat equation in the model in Eq (11) strongly depends on the gradient of the action potential. As shown in [21], this can be achieved using the Mitchell–Schaeffer model and, by tuning the parameters of this model, it is possible to obtain an action potential with nearly realistic amplitude, duration, and upstroke velocity. In this study, following the adjustment of the Mitchell–Schaeffer parameters, the suitable values presented in Table 1 provide an upstroke of ∼1 ms and a conduction velocity of ∼0.7 m/s at 37 °C. In addition, for both the Mitchell–Schaeffer and the Aliev–Panfilov models, we use a resting voltage of *V*_*rest*_ = −85 mV, a peak value of *V*_*peak*_ = 40 mV, and a total amplitude of the action potential as *V*_*amp*_ = *V*_*peak*_ − *V*_*rest*_, to obtain a dimensional version of the electrophysiological wave.

**Table 1:** Parameters used in the dimensional Mitchell–Schaeffer and Aliev–Panfilov models.

Mitchell–Schaeffer model		Aliev–Panfilov model	
constant	value	constant	value
*τ*_*in*_	0.05 (ms)	*k*	8
*τ*_*out*_	1 (ms)	*a*	0.15
*τ*_*open*_	95 (ms)	*u*_1_	0.2
*τ*_*close*_	162 (ms)	*u*_2_	0.3
*v*_*gate*_	0.13(mV)	*ϵ*_0_	0.002
		*V*_*a*_	*V*_*amp*_*a* + *V*_*rest*_(*mV*)

In the case of the bidomain tissue model, several values for the constants are available. In this study, we use the calibrated parameters obtained from [22] for the surface to volume ratio *χ*, the capacitance *C*_*m*_, and the conductances **G**_*i*_ and **G**_*e*_ (see Table 2). In [23], a full discussion of the experimentally measured values of the conductances can be found.

**Table 2:** Parameters used in the bidomain tissue model.

constants	values	units
*χ*	2000	*cm*^−1^
*C*_*m*_	1	*μF*/*cm*^2^
**G**_*i*_	$\left(\begin{array}{cc} 3 & 0\\ 0 & 0.315 \end{array}\right)$	*mS*/*cm*
**G**_*e*_	$\left(\begin{array}{cc} 2 & 0\\ 0 & 1.35 \end{array}\right)$	*mS*/*cm*

For the Pennes’ bioheat Eq (8), the available experimental data for the heat properties of cardiac tissue is limited [9]; however, the values of thermal conductivity *k*, density *ρ*, and heat capacity *c*_*p*_, were experimentally determined for cardiac muscle [24]. We were unable to find an equivalent experimental value for the metabolic and blood perfusion term *b*_*c*_ for cardiac tissue. Nevertheless, in [25] and [26], the heat due to the propagation of the electric wave in both the olfactory and the myelinated nerve fibers was rapidly reabsorbed by the medium (∼30 ms). Therefore, despite the possible differences in the behavior of myocytes and nerve cells, we performed a parametric study to reproduce this re-absorption as performed in [9]. The values used in Pennes’ bioheat equation are given in Table 3.

**Table 3:** Parameters used in Pennes’ bioheat equation.

constants	values	units
*ρ*	1.084 × 10^−3^	*kg*/*cm*^3^
*c*_*p*_	3676	*J*/(*kg* °*C*)
*k*	5.6 × 10^−6^	*J*/(*mscm* °*C*)
*b*_*c*_	8 × 10^−5^	*J*/(*mscm*^3^ °*C*)

As the Mitchell–Schaeffer and Aliev–Panfilov models have not been used previously to study temperature effects, constants *A*, *B*, and *Q* in Eqs (9) and (10) are not available in literature in the context of these cell models. The values chosen for the simulations, shown in Table 4, are within the range of the experimental values found in [11].

**Table 4:** Temperature parameters used in the models.

Mitchell–Schaeffer model		Aliev–Panfilov model	
constant	value	constant	value
*A*	1	*A*	1
*B*	0.07 (°*C*)^−1^	*B*	0.081 (°*C*)^−1^
*Q*	2.4	*Q*	2.4

## 3 Methods

### 3.1 Finite element discretization

A second-order mixed finite element formulation in both space and time was used for the bidomain model. For space discretization quadratic polynomials were used. A second-order fully implicit backward scheme (Gear method) was employed for the time derivative discretization. For instance, starting from *T*^*n*−1^ and *T*^*n*^, the Gear scheme gives

$$\begin{array}{c} \frac{\partial T}{\partial t}\left(t^{\left(n+1\right)}\right)≃\frac{3T^{\left(n+1\right)}-4T^{\left(n\right)}+T^{\left(n-1\right)}}{2\Delta t}. \end{array}$$

Using *ψ*_*v*_, *ψ*_*ϕ*_, *ψ*_*w*_, and *ψ*_*T*_ as test functions, the overall algorithm for solving the proposed heat–bidomain model on a fixed mesh is as follows.

1. Starting from the solutions at time *t*^*n*−1^ and *t*^*n*^, approximations $\left(V_{m}^{\left(n+1\right)},\phi_{e}^{\left(n+1\right)},W^{\left(n+1\right)}\right)$ are obtained based on the following system:
  $$\begin{array}{c} \left\{ \begin{array}{c} \int_{\Omega }\chi C_{m}\frac{3V_{m}^{\left(n+1\right)}-4V_{m}^{\left(n\right)}+V_{m}^{\left(n-1\right)}}{2\Delta t}\psi_{v}\;d\Omega +\int_{\Omega }G_{i}\nabla V_{m}^{\left(n+1\right)}\cdot \nabla \psi_{v}\;d\Omega \\ =-\int_{\Omega }G_{i}\nabla \phi_{e}^{\left(n+1\right)}\cdot \nabla \psi_{v}\;d\Omega -\int_{\Omega }\chi I\left(V_{m}^{\left(n+1\right)},W^{\left(n+1\right)},T^{\left(n\right)}\right)\psi_{v}\;d\Omega ,\\ -\int_{\Omega }\left(G_{i}+G_{e}\right)\nabla \phi_{e}^{\left(n+1\right)}\cdot \nabla \psi_{\phi }\;d\Omega =\int_{\Omega }G_{i}\nabla V_{m}^{\left(n+1\right)}\cdot \nabla \psi_{\phi }\;d\Omega ,\\ \int_{\Omega }\frac{3W^{\left(n+1\right)}-4W^{\left(n\right)}+W^{\left(n-1\right)}}{2\Delta t}\psi_{w}\;d\Omega =\int_{\Omega }F\left(V_{m}^{\left(n+1\right)},W^{\left(n+1\right)},T^{\left(n\right)}\right)\psi_{w}\;d\Omega , \end{array}\right. \end{array}$$ (16)
2. Starting from $V_{m}^{\left(n+1\right)}$, the approximation (*T*^(*n*+1)^) is obtained based on the following system:
  $$\begin{array}{c} \left\{ \begin{array}{c} \int_{\Omega }\rho c_{p}\frac{3T^{\left(n+1\right)}-4T^{\left(n\right)}+T^{\left(n-1\right)}}{2\Delta t}\psi_{T}\;d\Omega +\int_{\Omega }k\nabla T^{\left(n+1\right)}\cdot \nabla \psi_{T}\;d\Omega \\ =\int_{\Omega }b_{c}\left(T^{*}-T^{\left(n+1\right)}\right)\psi_{T}^{\left(n+1\right)}\;d\Omega +\int_{\Omega }\left(G_{i}\nabla V_{m}^{\left(n+1\right)}\cdot \nabla V_{m}^{\left(n+1\right)}\right)\psi_{T}\;d\Omega . \end{array}\right. \end{array}$$ (17)
3. Return to step (1).

At each time step, the Newton’s method was used to solve the non-linear system mentioned above. The linear systems resulting from the Newton’s method were solved by iterative methods using incomplete *LU* decomposition (ILU) generalized minimal residual (GMRES) solver [27] from the PETSc library [28].

### 3.2 Time-dependent adaptive algorithm

Although a second-order mixed finite element formulation method for both space and time was used, the above-mentioned algorithm presents complex computational challenges. In addition to computational difficulties related to the bidomain model, the source term in the Pennes’ bioheat Eq (8) strongly depends on the gradients of the action potential, and as the transmembrane potential is a traveling wave with a very sharp depolarization front in cardiac tissues, the numerical simulation is more intensive computationally and requires extremely fine meshes. Therefore, an accurate numerical method is necessary for suitable simulations of the temperature effect on the electrical waves in the human heart.

Mesh adaptative methods can be more suitable to address these difficulties. The main advantage of these methods is that finer mesh cells can be located near the front, while a coarser mesh can be used away from the front. Therefore, the accuracy of the prediction of the electrical wavefronts is enhanced, and the total number of mesh elements can be greatly reduced, along with the computational time. Several adaptive strategies have been introduced in the context of simulating the cardiac electrical activity (see [29], [30], [31], [32] and the references therein). An adaptive method, based on a hierarchical error estimator, was developed for the two-dimensional simulation of electrical waves in the heart [33]. A three-dimensional adaptive method, based on the definition of edge lengths using a solution-dependent metric, was employed in [34]. In this work, we adopt an adaptive mesh technique based on the definition of edge lengths using a solution-dependent metric. A complete description of this technique is presented in [34, 35] and not repeated here. The computational efficiency of our adaptive algorithm was previously assessed on single and complex cardiac wave dynamics [36]. However, the heat–bidomain model presented in this paper requires a different time-dependent algorithm which is described in the following.

The overall adaptive algorithm has the following steps:

1. Start from the solutions *T*^(*n*)^, $V_{m}^{\left(n-1\right)}$, $V_{m}^{\left(n\right)}$, *W*^(*n*−1)^, *W*^(*n*)^, $\phi_{e}^{\left(n-1\right)}$, and $\phi_{e}^{\left(n\right)}$ and a mesh $M^{\left(n\right)}$ at time *t*^(*n*)^;
  time *t*^(*n*)^;
2. Solve the system in Eq (16) on mesh $M^{\left(n\right)}$ to obtain a first approximation of the solutions (denoted by ${\tilde{V_{m}}}^{\left(n+1\right)}$, ${\tilde{W}}^{\left(n+1\right)}$ and ${\tilde{\phi_{e}}}^{\left(n+1\right)}$) at time *t*^(*n*+1)^;
3. Start from the solutions ${\tilde{V_{m}}}^{\left(n+1\right)}$, *T*^(*n*−1)^, *T*^(*n*)^ and the mesh $M^{\left(n\right)}$ at time *t*^(*n*)^;
  time *t*^(*n*)^;
4. Solve the system in Eq (17) on mesh $M^{\left(n\right)}$ to obtain a first approximation of the solution (denoted by ${\tilde{T}}^{\left(n+1\right)}$) at time *t*^(*n* + 1)^;
5. Adapt the mesh starting from mesh $M^{\left(n\right)}$ and the solution-dependent metric calculated from the solutions $V_{m}^{\left(n-1\right)}$, $V_{m}^{\left(n\right)}$, ${\tilde{V_{m}}}^{\left(n+1\right)}$, *W*^(*n*−1)^, *W*^(*n*)^, ${\tilde{W}}^{\left(n+1\right)}$, $\phi_{e}^{\left(n-1\right)}$, $\phi_{e}^{\left(n\right)}$, ${\tilde{\phi_{e}}}^{\left(n+1\right)}$, *T*^(*n*−1)^, *T*^(*n*)^, and ${\tilde{T}}^{\left(n+1\right)}$ to obtain a new mesh $M^{\left(n+1\right)}$;
6. Reinterpolate $V_{m}^{\left(n-1\right)}$, $V_{m}^{\left(n\right)}$, *W*^(*n*−1)^, *W*^(*n*)^, $\phi_{e}^{\left(n-1\right)}$, $\phi_{e}^{\left(n\right)}$, *T*^(*n*−1)^, and *T*^(*n*)^ on the mesh $M^{\left(n+1\right)}$;
7. Solve the system in Eq (16) on the mesh $M^{\left(n+1\right)}$ for $V_{m}^{n+1}$, *W*^*n*+1^, and ${\phi_{e}}^{n+1}$.
8. Solve the system in Eq (17) on the mesh $M^{\left(n+1\right)}$ for *T*^*n*+1^.
9. Next time step: go to step 2.

According to step 5, adapting the mesh using all different numerical solutions at each time step is more favorable, which depends on the time discretization scheme. In this work, a second-order fully implicit backward scheme was employed for the time stepping. Thus, the mesh is required to represent the solutions at times *t*^(*n*−1)^, *t*^(*n*)^, and *t*^(*n*+1)^. We adopted a common metric on which the mesh is adapted by considering the errors on all these solutions. Moreover step 5 indicates that 12 variables need to be considered. This is costly and therefore to reduce the computational time, a linear combination of the different solutions can be adapted. In our experience, the following four variables provide satisfactory results:

$$\begin{array}{c} \frac{V_{m}^{\left(n-1\right)}+V_{m}^{\left(n\right)}+{\tilde{V_{m}}}^{\left(n+1\right)}}{3},\frac{W^{\left(n-1\right)}+W^{\left(n\right)}+{\tilde{W}}^{\left(n+1\right)}}{3}\text{,}\frac{\phi_{e}^{\left(n-1\right)}+\phi_{e}^{\left(n\right)}+{\tilde{\phi_{e}}}^{\left(n+1\right)}}{3}\;\text{and}\;\frac{T^{\left(n-1\right)}+T^{\left(n\right)}+{\tilde{T}}^{\left(n+1\right)}}{3}. \end{array}$$

## 4 Results

In this section, we discuss the performance of the model and the numerical method introduced in the previous sections. First, we present the effect of heat on regular electrophysiological wave. The cardiac transmembrane potential is demonstrated at different temperatures and the results are validated by comparing the calculated and the experimental values of *Q*_10_, which is a commonly used quantification in biology to describe the rate of change of a system subjected to a temperature increase of 10 °C. Then, the effect of hypothermia in suppressing cardiac arrhythmias is investigated.

### 4.1 Effect of heat on action potential duration and conduction velocity

In this section, a two-dimensional regular wave is presented. The simulation was performed for 750 ms on a sheet of size of 10 cm × 10 cm. The heat–bidomain model combined with the Mitchell–Schaeffer model was used for the ionic representation. All physical parameters are summarized in Tables 1, 2 and 3. Homogeneous Neumann conditions were applied on all sides as boundary condition, with the following initial conditions:

$$\begin{array}{cc} & V_{m}=\{\begin{array}{cc} 40 & \sqrt{{\left(x-5\right)}^{2}+{\left(y-5\right)}^{2}}<0.5,\\ -85 & \text{otherwise,} \end{array} \end{array}$$

$$\begin{array}{cc} & \phi_{e}=0,\;W=0.9.\; \end{array}$$

**Fig 1:**
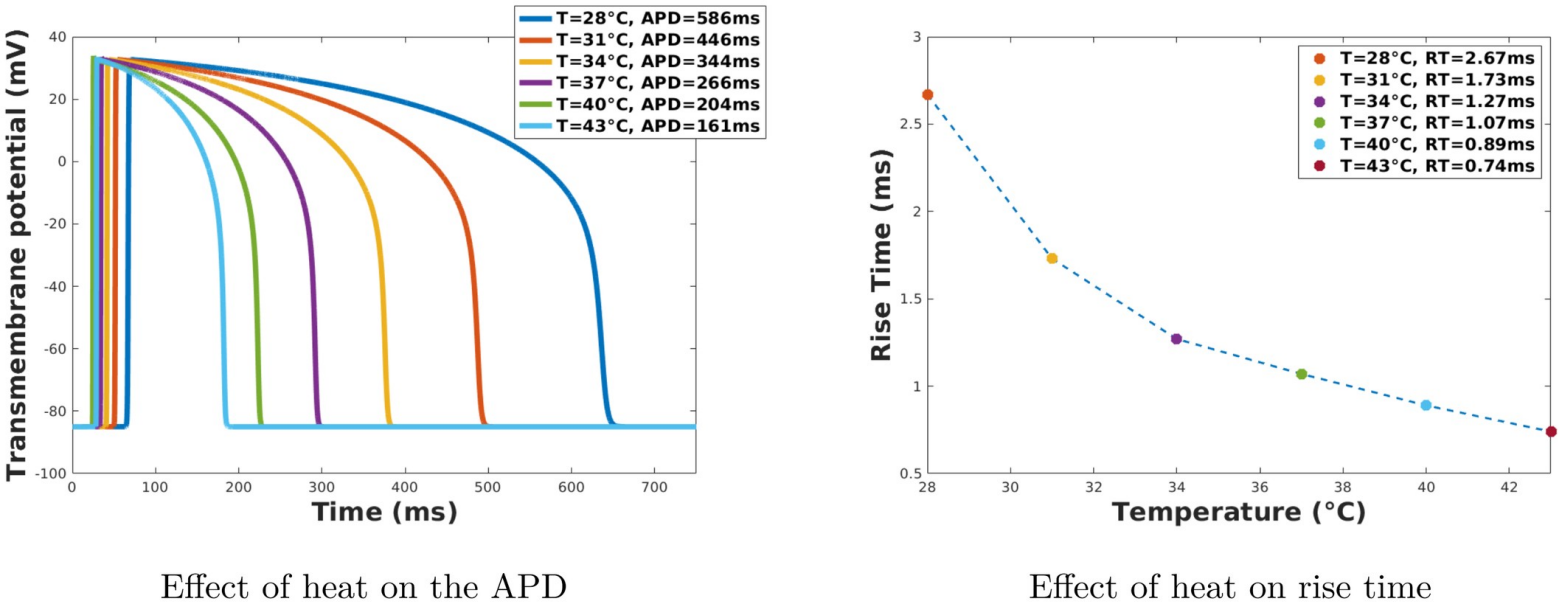
Effect of heat on APD and rise time. The APDs were calculated as the duration at which the voltage is maintained above −80 mV at point (6, 5). The rise time is calculated as the time interval at which the action potential increases from −80 mV to 30 mV.

Six different values were considered for T*: 28, 31, 34, 37, 40, and 43 °C. The initial temperature was set to be equal to the value of T*. A time step of *t* = 0.3 ms was employed in all calculations. This time step was relatively large because a fully implicit method combined with an adaptive mesh technique was used. However, a smaller time step is be required when using regular meshes or at T* = 43 °C, where the wavefront is sharper. The effects of these changes in temperature on the APD and the rise time is shown in Fig 1. As can be seen in Fig 1a), the increase in temperature significantly decreases the APD, while a decrease in temperature increases it. This is the expected non-linear behavior, compared to the experimental results presented in [37]. Fig 1b) shows that the rise time decreases when the temperature is increased.

Several studies were performed using the *Q*_10_ temperature coefficient. The *Q*_10_ value can be used to describe the temperature sensitivity of properties in cardiac tissue, such as the temperature dependence of the APD and the conduction velocity. The experimental value of *Q*_10_(*CV*) = 2.3 was determined in rat cardiomyocytes [38] and a value of *Q*_10_(*APD*) = 2.5 [39] was determined through measurements of guinea pig ventricular myocytes. Using the results of our simulations, it is possible to measure these values based on the following equations:

$$\begin{array}{c} Q_{10}\left(APD\right)=(\frac{APD_{27\;{}^{\circ}\text{C}}}{APD_{37\;{}^{\circ}\text{C}}}{)}^{10/(37\;{}^{\circ}\text{C}-27\;{}^{\circ}\text{C})} \end{array}$$

$$\begin{array}{c} Q_{10}\left(CV\right)=(\frac{CV_{27\;{}^{\circ}\text{C}}}{CV_{37\;{}^{\circ}\text{C}}}{)}^{10/(27\;{}^{\circ}\text{C}-37\;{}^{\circ}\text{C})} \end{array}$$

The APDs were calculated as the duration at which the voltage is maintained above −80 mV at point (6, 5). Additionally, the conduction velocity was calculated using the time difference between the first front crossing −80 mV at locations near (6, 5). Table 5 shows the obtained calculated values, which are in good agreement with the experimentally measured values in [38] and [39].

**Table 5:** Measured APD, CV, and *Q*_10_ values from the simulations.

	T* = 37°*C*	T* = 27°*C*	*Q*_10_ measured
*APD*(*ms*)	266	644	2.42
*CV*(*m*/*s*)	0.741	0.337	2.19

**Fig 2:**
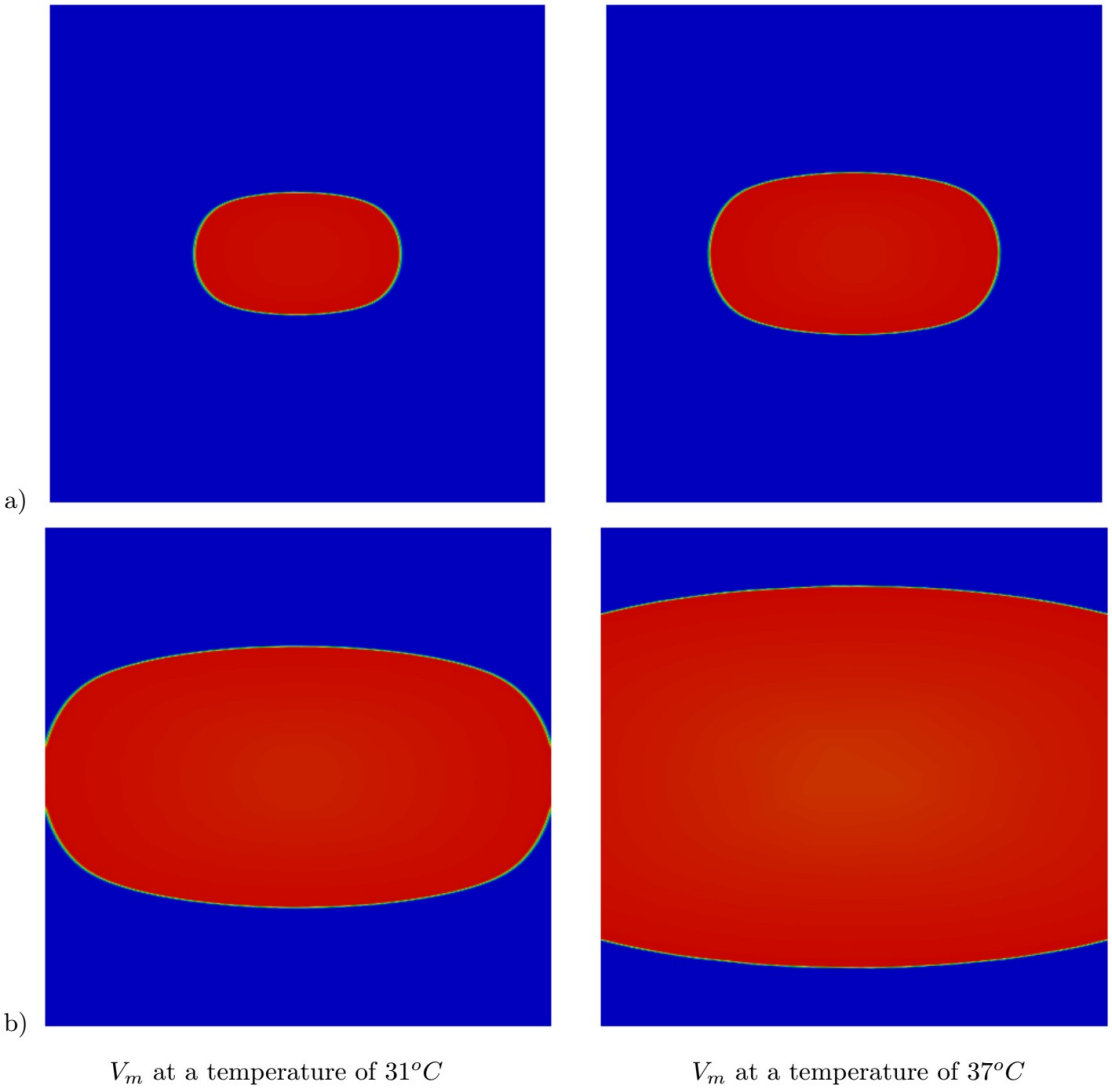
Time evolution of the transmembrane potential *V*_*m*_. Results at a) *t* = 33 ms and b) *t* = 93 ms. Numerical results at two different temperatures: T* = 31 °C (first column) and at T* = 37 °C (second column).

**Fig 3:**
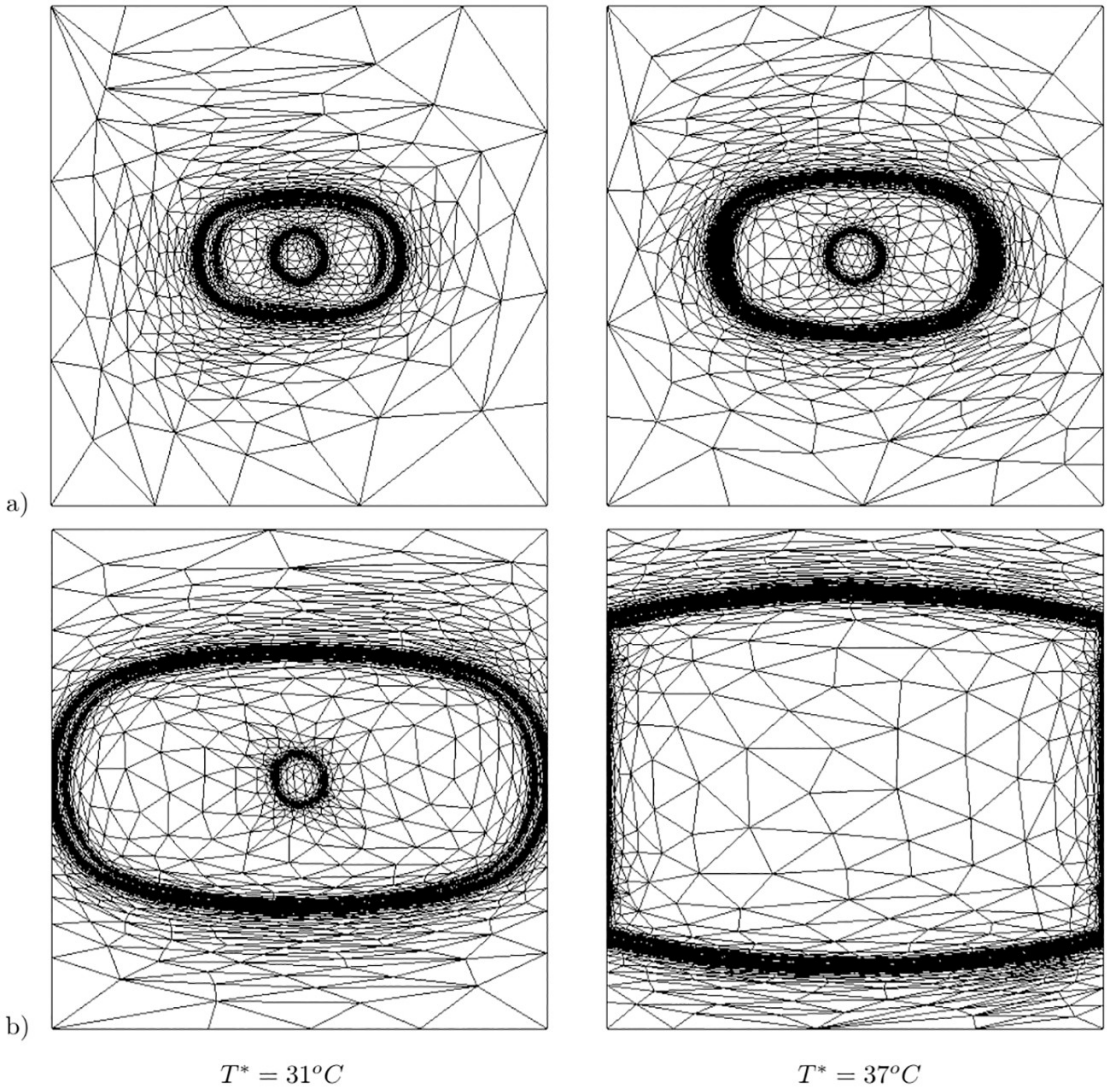
Time evolution of adapted mesh. Results at a) *t* = 33 ms and b) *t* = 93 ms. Numerical results at two different temperatures: T* = 31 °C (first column) and at T* = 37 °C (second column).

Furthermore, owing to the inclusion of Pennes’ bioheat equation to the bidomain model, it is possible to study the effect of heat on the electrical wave propagation. Fig 2 shows the transmembrane potentials at T* = 31 °C and T* = 37 °C obtained at a single time instant. As can be seen in the figure, the area of the excited potential is larger for the warmer tissue. This is due to the higher propagation speed at higher temperatures. For these simulations, the Mitchell–Schaeffer model was employed, and at 37 °C, the applied parameters result in a conduction velocity of ∼0.7 ms and a rise time of 1.07 ms. Higher upstroke and conduction velocities were obtained when the temperature was increased; thus, this model is suitable to demonstrate the performance of the presented adaptive strategy. Fig 3 shows these adapted meshes at different time instants for both T* = 31 °C and T* = 37 °C. This demonstrates that elongated elements are obtained at the appropriate position to capture the transmembrane potential wave. The adapted mesh evolves with time, and at each time step, the adapted meshes are obtained to accurately determine the stiffness of the depolarization wavefront, which enables the feasibility of the technique for this study.

### 4.2 Effect of hypothermia in suppressing cardiac arrhythmias

**Fig 4:**
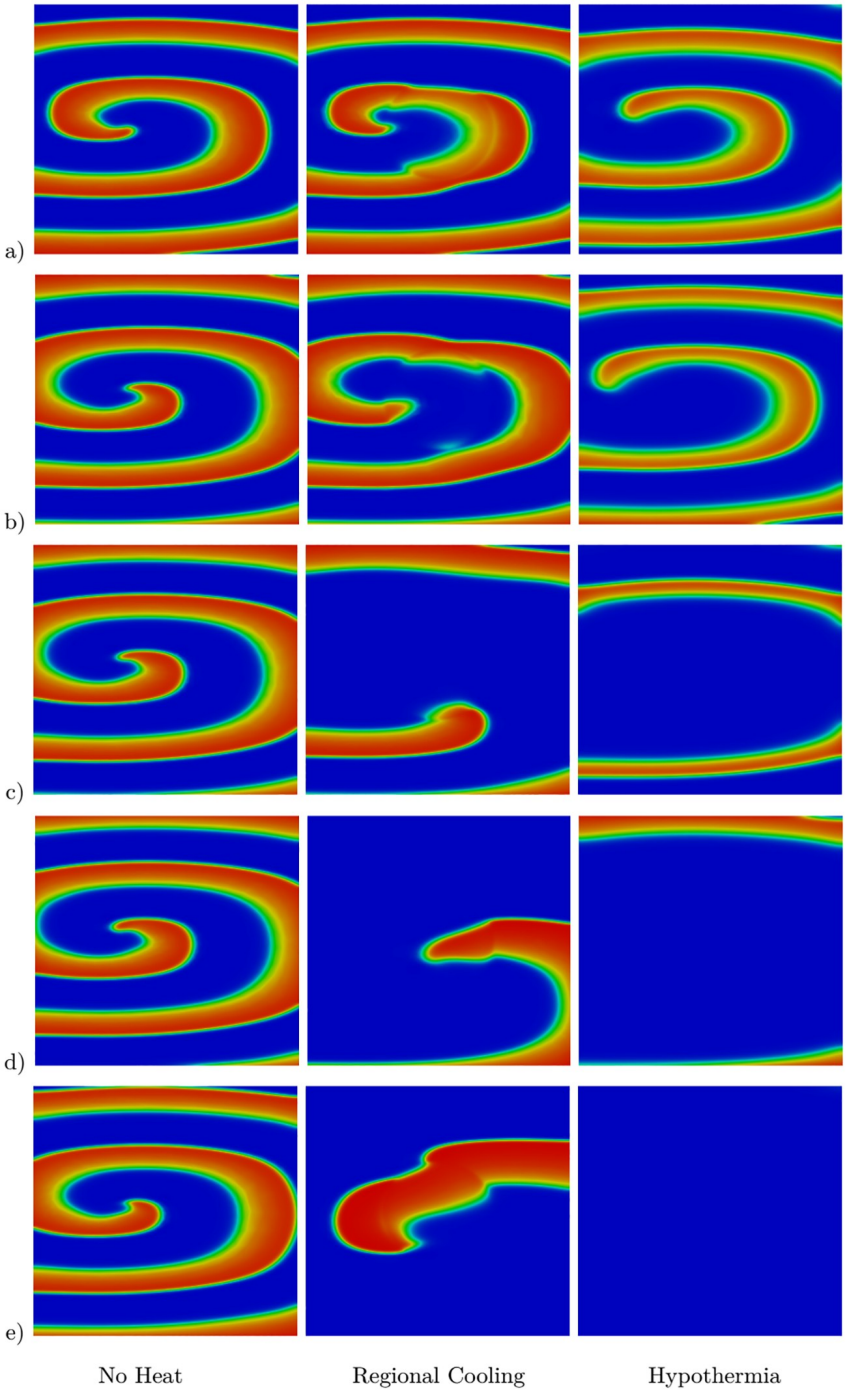
Effect of heat on a single spiral wave at five different time instances. Results at a) *t* = 300 ms, b) *t* = 450 ms, c) *t* = 900 ms, d) *t* = 1350 ms, and e) *t* = 1680 ms. The first column shows results at body temperature (T* = 37 °C in the tissue), the second column shows the case of a regional cooling (T* = 30 °C in the center of the tissue), and the third column considers the case of hypothermia (T* = 30 °C in the tissue).

One of the main advantages of the proposed model and the methodology used in this study is that it enables the investigation of the effect of hypothermia and regional cooling on cardiac tissue. For this purpose, we first study a single spiral wave generated by a similar technique described in [36] and with an initial temperature of 37 °C. We consider the heat–bidomain model combined with the Aliev–Panfilov ionic model. All physical parameters are given in Tables 1, 2 and 3 except for *a*, which has a value of 0.1. The regional cooling is generated by the source term in the model in Eq (11). In particular, we set T* to 30 °C in a circle of radius of 2 cm centered in the computational domain, while 37 °C is maintained in the rest of the domain. The case of hypothermia is considered by setting T* to 30 °C in the entire computational domain. The results are shown in Fig 4. The first column in this figure shows the transmembrane potential when there is no heat effect (T* = 37 °C), the effect of regional cooling is presented in the second column, and the last column shows the case of hypothermia. As can be seen, the regional cooling prolongs the transmembrane potential, but it does not suppress the spiral wave. However, the hypothermia at T* = 30 °C allows the termination of the spiral during the first 1.68 s interval of the simulation.

**Fig 5:**
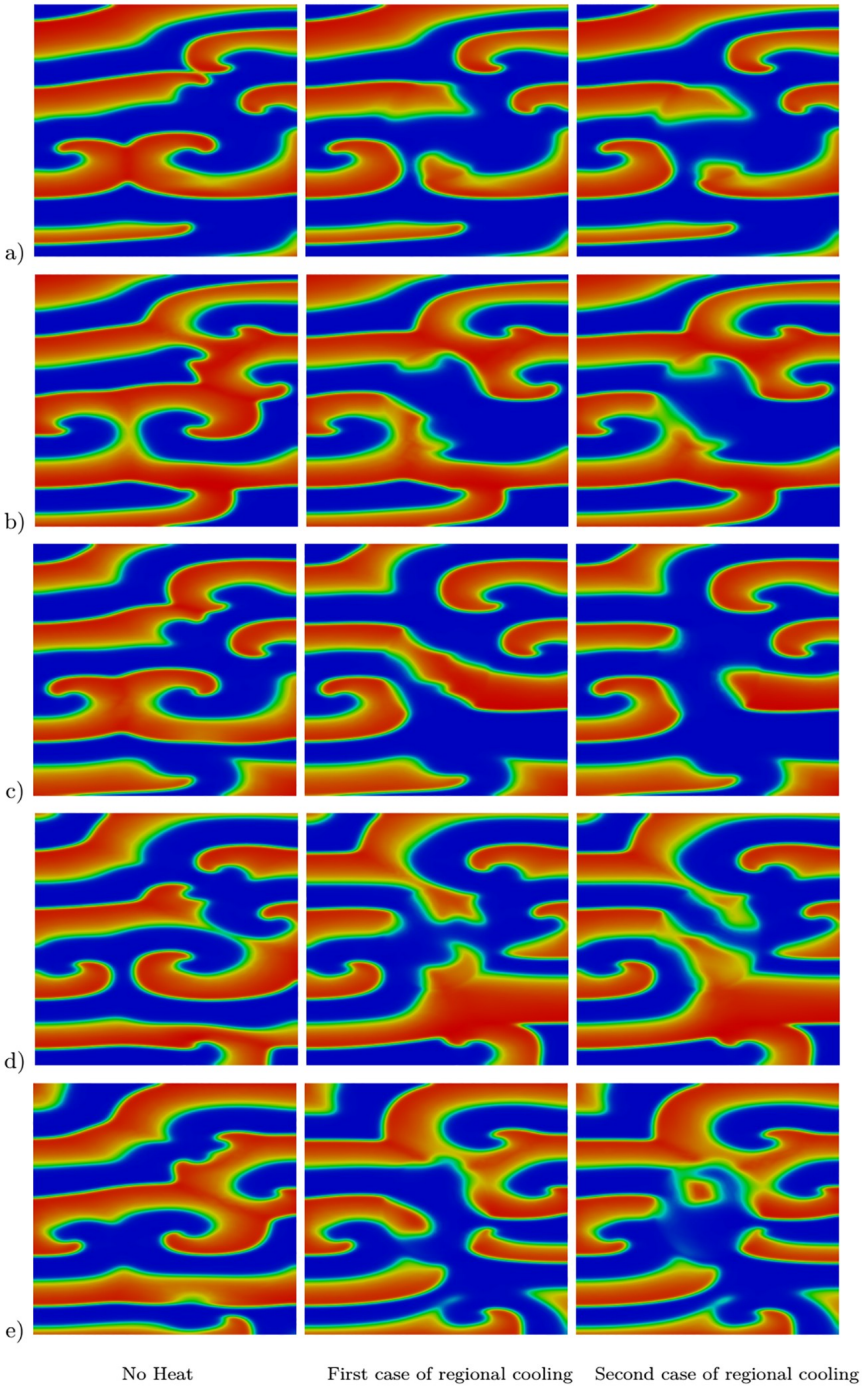
Effect of heat on multiple spiral waves at five different time instances. Results at a) *t* = 900 ms, b) *t* = 1050 ms, c) *t* = 1350 ms, d) *t* = 1650 ms, and e) *t* = 1950 ms. The first column shows results at body temperature (T* = 37 °C in the tissue), the second column shows the results for the first case of regional cooling (T* = 30 °C in the center of the tissue), and the third column shows the results of the second case of regional cooling (T* = 28 °C in the center of the tissue).

**Fig 6:**
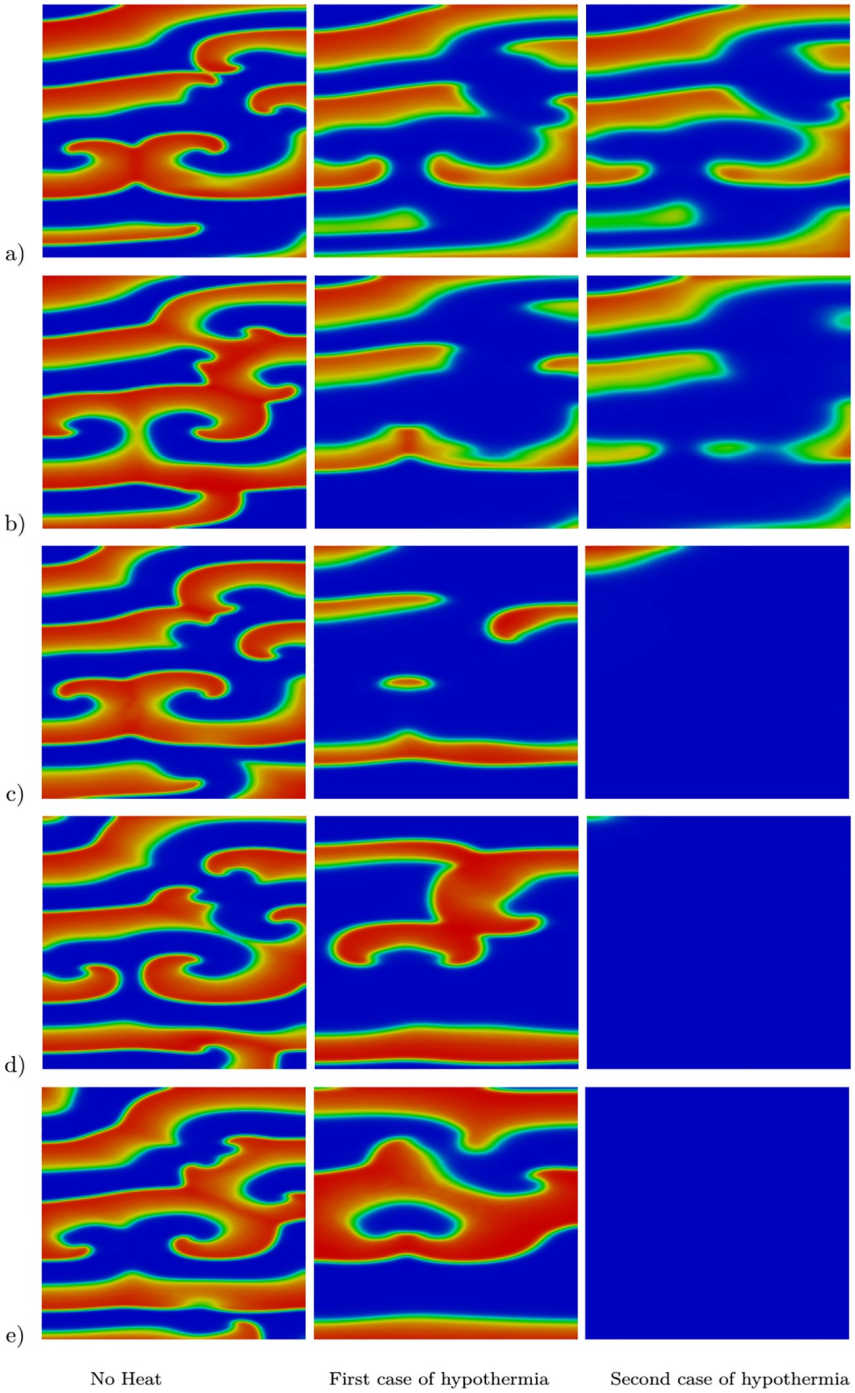
Effect of heat on multiple spiral waves at five different time instants. Results at a) *t* = 900 ms, b) *t* = 1050 ms, c) *t* = 1350 ms, d) *t* = 1650 ms, and e) *t* = 1950 ms. The first column shows results at body temperature (T* = 37 °C in the tissue), the second column shows the results of the first case of hypothermia (T* = 30 °C in the tissue), and the third column shows the results of the second case of hypothermia (T* = 28 °C in the tissue).

These results were investigated in more complex spiral waves as well. The study begun with three spiral waves and then the parameters of the Aliev–Panfilov model were set to the values *a* = 0.1, *μ*_1_ = 0.135, and *ϵ* = 0.001. The initial temperature and the value of T* were set to 37 °C. After *t* = 600 ms we considered two cases of regional cooling, where T* was set to either 30 °C or 28 °C in a circle of radius of 2 cm centered in the computational domain, while 37 °C was maintained in the rest of the domain, and two cases of global hypothermia, where T* was set to either 30 °C or T* = 28 °C in the entire computational domain. The transmembrane potential in the case of regional cooling is shown in Fig 5, where the first column shows the transmembrane potential in the case of no temperature variation (T* = 37 °C), the effect of regional cooling using 30 °C is presented in the second column, and the last column shows the effect of regional cooling at 28 °C. Similarly, the transmembrane potential in the case of hypothermia is shown in Fig 6, where the first column shows the transmembrane potential in the case of no heat effect (T* = 37 °C), the first case of hypothermia (T* = 30 °C) is presented in the second column, while the last column shows the second case of hypothermia (T* = 28 °C). As can be seen, in the case of regional cooling (Fig 5) the spiral waves are not terminated and they are breaking up into multiple spirals, which can be considered pro-arrhythmic. However, hypothermia at T* = 30 °C promotes the prolongation of waves and reduces the number of spirals, while hypothermia at T* = 28 °C terminates all spirals during the first 1.35 s interval of the simulations.

**Fig 7:**
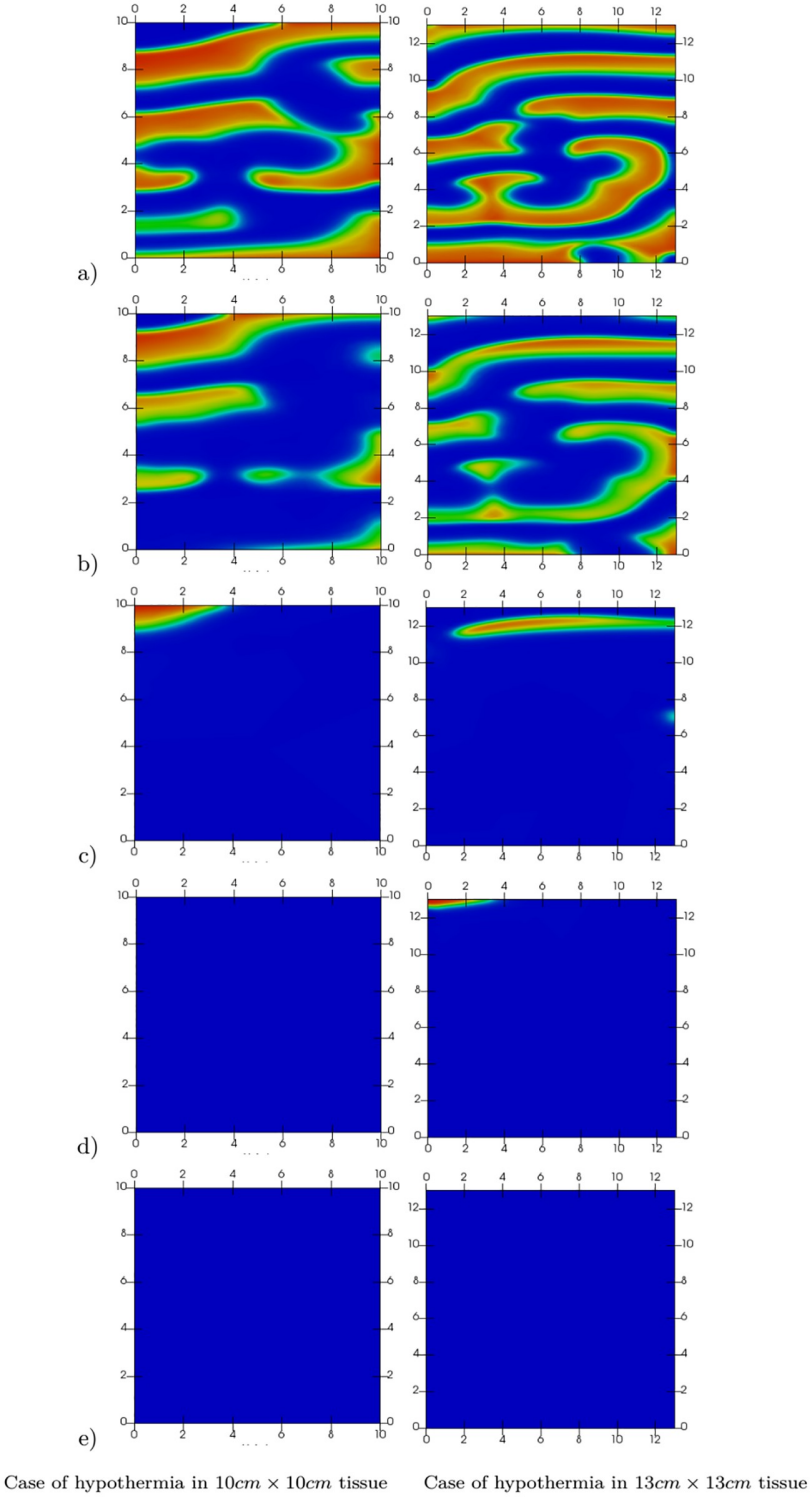
Effect of hypothermia (T* = 28 °C) on multiple spiral waves in two different tissue sizes. Results at a) *t* = 900 ms, b) *t* = 1050 ms, c) *t* = 1450 ms, d) *t* = 1950 ms, and e) *t* = 2550 ms. The first column shows results for the region of 10 cm × 10 cm and second column shows results for the region of 13 cm × 13 cm.

To investigate if the spiral wave was terminated due to the physical boundary, we performed simulations in a region of 13 cm × 13 cm. As shown in Fig 7, the hypothermia can still suppress the spiral waves in this larger computational domain. Based on our numerical simulations, the spiral wave can remain in the tissue depending on the value of the cooling temperature and independent of the size of the tissue. In the case of one spiral wave, a temperature of 30 °C was sufficient to suppress the wave; however, for a more complex case, a temperature of 28 °C was required to terminate the waves.

**Fig 8:**
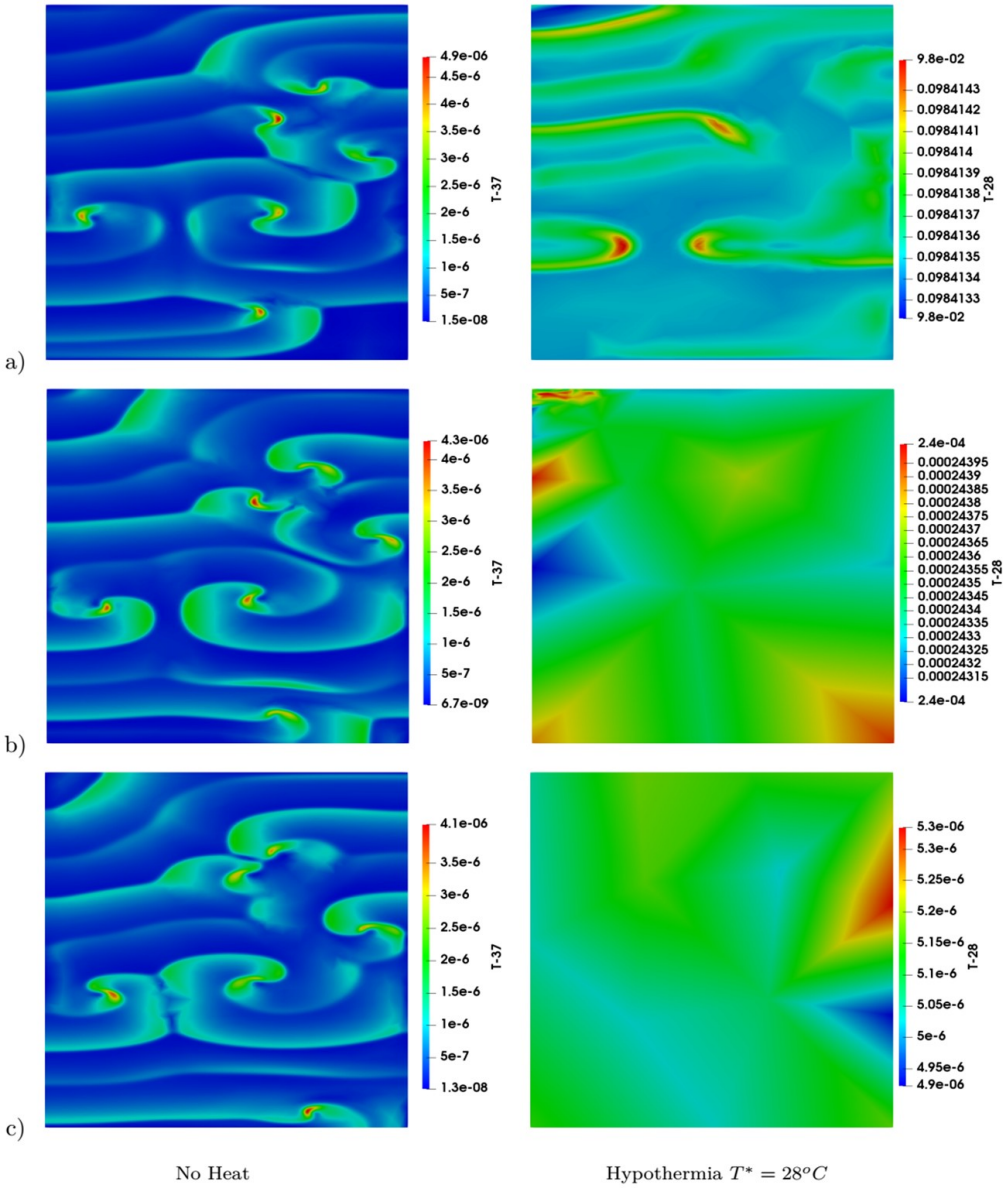
Joule effect on temperature in the case of multi-spiral waves. Results at a) *t* = 900 ms, b) *t* = 1450 ms, and c) *t* = 2550 ms. The first column shows results without heat (T* = 37 °C in the tissue) and the second column shows the case of hypothermia (T* = 28 °C).

The heat induced by the Joule effect associated with the action potential is in the order of magnitude of *μ*°C. Although this change is small, it is of similar magnitude as that previously found in nerves and intestinal tissues [8–10]. It was also found, that the heat due to the Joule effect accumulated in these tissues at the spiral tip; therefore, it could be potentially used as an alternative method for detecting the location of the spiral waves tips. Our simulations showed similar results for the cardiac tissue. Fig 8 shows the temperature field, *T* − T*. As can be seen, that in the case of no heat (T* = 30 °C), as the spiral wave evolves, an accumulation of heat develops near the tip of the spiral wave (shown in the first column in Fig 8). However, this behavior is not present in the case of hypothermia, (T* = 28 °C), where the temperature in the tissue is nearly constant in the entire computational domain (shown in the second column in Fig 8). The accumulation of heat was not observed in the case of hypothermia, which provides a potential explanation for the inhibition of spiral waves formation and for the promotion of termination of these waves by hypothermia.

**Fig 9:**
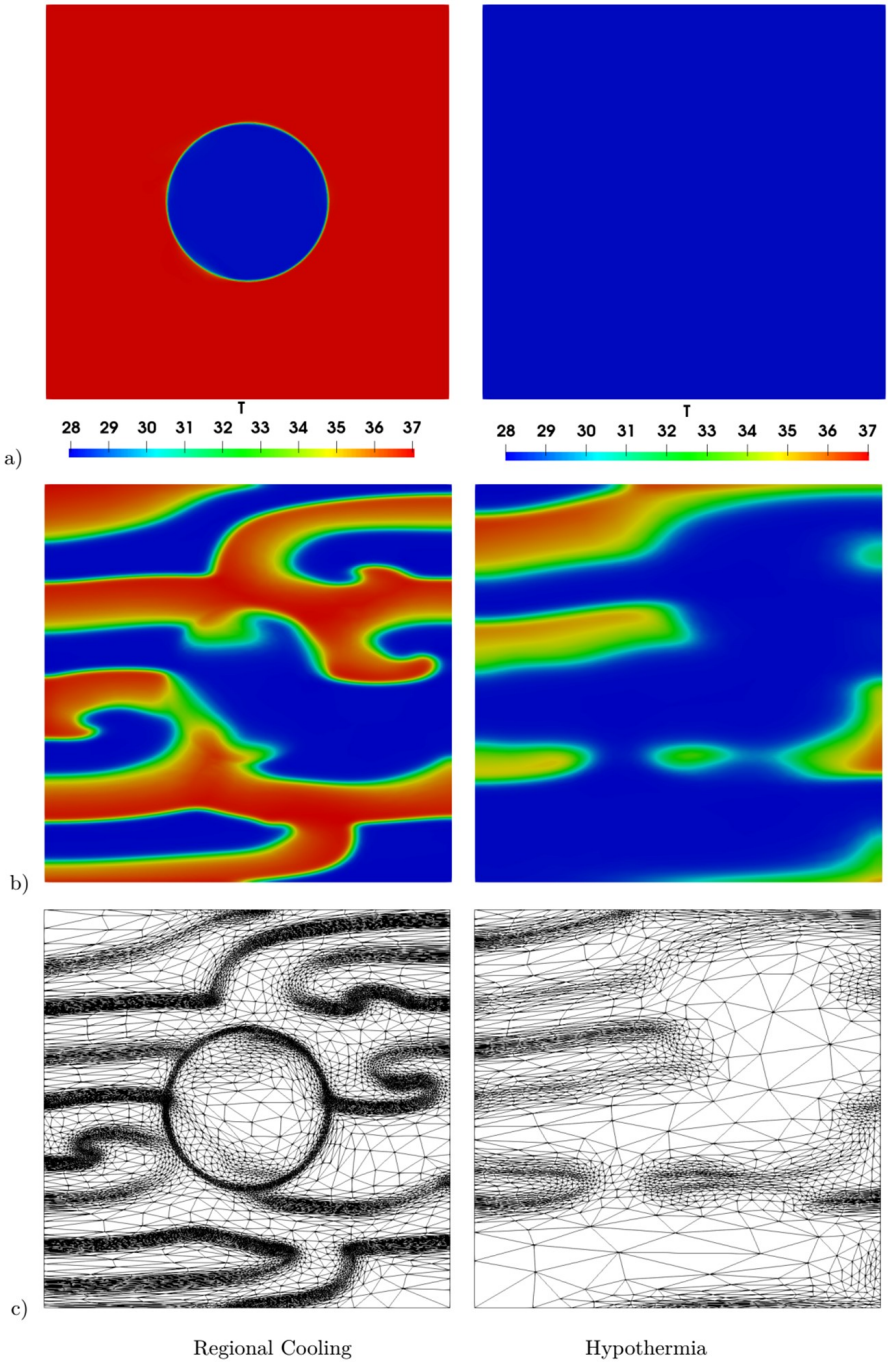
Adapted mesh and the corresponding solutions for both regional cooling (first column) and hypothermia (second column). a) Temperature *T*, b) transmembrane potential *V*_*m*_, and c) the corresponding adapted mesh.

All numerical results were obtained using the adaptive method described in section 3.2. The obtained meshes in the case of regional cooling and hypothermia are shown in Fig 9. As can be seen, using the adaptive mesh, accurate numerical results can be obtained. In the case of regional cooling (first column), the mesh is well adapted not only around the de- and repolarization fronts but also around the circle where *T* is set as 30 °C. Similarly, in the case of hypothermia (second column), the adapted mesh provides noticeably better results, as only few elements are obtained after the termination of the spirals waves.

**Fig 10:**
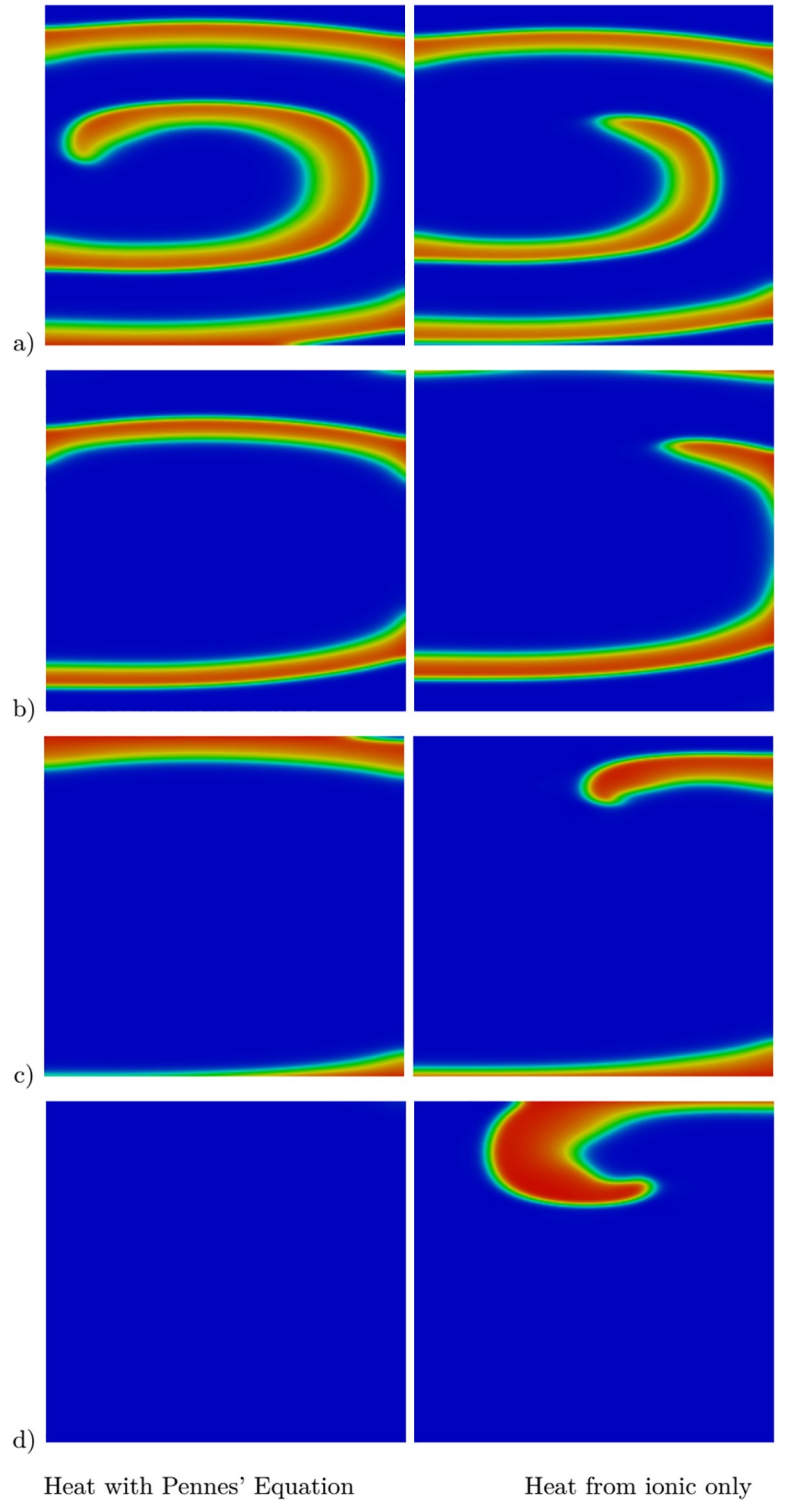
Effect of hypothermia (T* = 30 °C in the tissue) on a single spiral wave at five different time instances. Results at a) *t* = 450 ms, b) *t* = 900 ms, c) *t* = 1350 ms, and d) *t* = 1680 ms. The first column shows results for the proposed heat–bidomain model and the second column shows the results when heat is induced via the ionic model only.

As mentioned in the introduction, certain results in the literature include heat in the monodomain model via the ionic term only. Finally, we performed a comparison between the approach in the literature and the approach developed in our work. We simply replaced *T* by T* in Eqs (9) and (10). Then, the Pennes’ bioheat equation does not affect the bidomain model and the temperature is included via the ionic term only. We repeated the simulation for the case of hypothermia for one spiral wave, as shown in Fig 4, and we compared it to the case where *T* was replaced by T* in the ionic model. The simulations are illustrated in Fig 10. As can be seen, there are considerable differences between the two methods as the approach in the literature failed to terminate the spiral wave. Although our approach can be relatively more expensive computationally, it can still be considered more logical, as it considers the heat over the entire cardiac tissue rather than just the ionic terms. However, experimental results are needed to further validate the numerical results.

## 5 Conclusions and discussion

A mathematical model obtained by coupling Pennes’ bioheat equation with the bidomain model is presented to simulate the effects of temperature variations on the cardiac APD. The simplification of the proposed heat–bidomain model to the heat–monodomain model is also presented. Several numerical experiments were performed. We demonstrated the effect of temperature on the APD and the conduction velocity, and the obtained numerical results were compared to the experimental results in the literature. We also investigated the effects of hypothermia and regional cooling in suppressing cardiac arrhythmias. We clearly demonstrated that hypothermia of short duration could terminate the spiral wave breakup. To our knowledge, this is the first study that presents numerical modeling of the effect of hypothermia in cardiac arrhythmias. As mentioned in the introduction, the effect of temperature has been included in studies in the literature via the ionic terms. However, in our approach the effect of spatial temperature on the cardiac electrical activity has been introduced directly. The comparison between the two approaches presented in this paper demonstrates the advantages of the proposed methodology.

The heat generated by the Joule effect was shown to accumulate at the tips of the spiral waves under non-hypothermic conditions. Although this induced variation in temperature is very small (∼ *μ* °C), such small temperature changes have previously been detected using thin-film synthetic pyroelectric material, polyvinylidene fluoride (PVDF), when the heat produced by electrical propagation in myelinated nerve fibers was measured [26]. Therefore, there is a potential to apply this heat-accumulation property as an alternative method for detecting the location of spiral tips in cardiac tissues.

The coupling between the heat and the bidomain model depends on the gradient of the transmembrane potential, which results in challenges in the simulation. Therefore, an appropriate numerical method is necessary for simulating the developed heat–bidomain model. In our simulations we used a time adaptive mesh algorithm, which is suitable for this coupling. However, any other appropriate numerical method can be used for simulating the developed heat–bidomain model, including parallel computing on fixed meshes, high-order methods, etc.

The overall methodology presented in this paper can be extended to further consider important temperature effects on cardiac tissues, such as the heat generated by ionic pumps and mechanical contraction. In this paper, a Mitchell–Schaeffer ionic model is used, which provides a fast upstroke and reproduces similar behavior to the ventricular action potential. However, more complex cardiac ionic models can be included to the proposed heat–bidomain model to study the heat generated by ionic pumps that maintain ionic gradients crucial for the action potential. Preliminary results were obtained, where the heat–monodomain model was coupled to the Luo–Rudy model [20]. However, this type of coupled model requires different numerical algorithms than the one used in our study, which are in the focus of a future work. Heat generation by mechanical contraction is also important. However, it requires more complex coupling where the heat–bidomain model needs to be coupled with a large-deformation mechanical model. This is the subject of a future work, where our aim is to study the effects of temperature and contraction on spiral wave breakup as well.
